# Positive Selection in the Chromosome 16 *VKORC1* Genomic Region Has Contributed to the Variability of Anticoagulant Response in Humans

**DOI:** 10.1371/journal.pone.0053049

**Published:** 2012-12-28

**Authors:** Blandine Patillon, Pierre Luisi, Hélène Blanché, Etienne Patin, Howard M. Cann, Emmanuelle Génin, Audrey Sabbagh

**Affiliations:** 1 Inserm UMRS-946, Genetic Variability and Human Diseases, Institut Universitaire d’Hématologie, Université Paris Diderot, Paris, France; 2 Université Paris Sud, Kremlin-Bicêtre, France; 3 Institute of Evolutionary Biology, CEXS-UPF-PRBB, Catalonia, Barcelona, Spain; 4 Fondation Jean-Dausset-CEPH, Paris, France; 5 Human Evolutionary Genetics, CNRS URA3012, Institut Pasteur, Paris, France; 6 UMR IRD 216, Université Paris Descartes, Paris, France; Centers for Disease Control and Prevention, United States of America

## Abstract

*VKORC1* (vitamin K epoxide reductase complex subunit 1, 16p11.2) is the main genetic determinant of human response to oral anticoagulants of antivitamin K type (AVK). This gene was recently suggested to be a putative target of positive selection in East Asian populations. In this study, we genotyped the HGDP-CEPH Panel for six *VKORC1* SNPs and downloaded chromosome 16 genotypes from the HGDP-CEPH database in order to characterize the geographic distribution of footprints of positive selection within and around this locus. A unique *VKORC1* haplotype carrying the promoter mutation associated with AVK sensitivity showed especially high frequencies in all the 17 HGDP-CEPH East Asian population samples. *VKORC1* and 24 neighboring genes were found to lie in a 505 kb region of strong linkage disequilibrium in these populations. Patterns of allele frequency differentiation and haplotype structure suggest that this genomic region has been submitted to a near complete selective sweep in all East Asian populations and only in this geographic area. The most extreme scores of the different selection tests are found within a smaller 45 kb region that contains *VKORC1* and three other genes (*BCKDK, MYST1 (KAT8)*, *and PRSS8)* with different functions. Because of the strong linkage disequilibrium, it is not possible to determine if *VKORC1* or one of the three other genes is the target of this strong positive selection that could explain present-day differences among human populations in AVK dose requirement. Our results show that the extended region surrounding a presumable single target of positive selection should be analyzed for genetic variation in a wide range of genetically diverse populations in order to account for other neighboring and confounding selective events and the hitchhiking effect.

## Introduction

Oral anticoagulants of antivitamin K type (AVK) − such as warfarin and acenocoumarol − are widely prescribed drugs for the prevention and treatment of arterial and venous thromboembolic disorders [1], [2]. They exert their anticoagulant effect by inhibiting the vitamin K 2,3-epoxide reductase complex 1 (VKORC1). Besides well-known physiopathological and environmental factors, including age, sex, body mass index, disease states, co-medications and diet, genetic factors have been identified as major determinants of AVK dose variability [3]. Candidate-gene and genome-wide association studies have identified four main genes − *CYP2C9*, *CYP4F2*, *CYP2C18* and *VKORC1*− which explain together between 28.2% and 43.5% of the AVK dose variance [3], [4], [5], [6], [7]. *CYP2C9, CYP4F2* and *CYP2C18* encode proteins involved in the hepatic metabolism of AVK [8], [9], [10]. *VKORC1* encodes the VKORC1 enzyme, which is the direct pharmacologic target of AVK [11], [12]. Differences in the worldwide distribution of the most important polymorphisms influencing AVK dosing are likely to underlie the wide interethnic variability in AVK dose requirements: current population-based trends in warfarin dosing, as reported by the International Warfarin Pharmacogenetics Consortium, indicate a mean weekly dose of 21 mg in Asians, 31.5 mg in Europeans and 40 mg in individuals of African ancestry [13].

Recently, Ross *et al*. [14] documented the distribution of four functional variants located in the three main genes known to influence AVK dose requirement − rs9923231 (*VKORC1*), rs1799853 and rs1057910 (*CYP2C9*), and rs2108622 (*CYP4F2*) − in a large set of samples from the Human Genome Diversity Project - Centre d’Etude du Polymorphisme Humain (HGDP-CEPH) Panel, representing 52 world populations [15]. They observed a pattern of genetic differentiation among human populations for the *VKORC1* single nucleotide polymorphism (SNP) rs9923231. They applied three formal tests of positive selection to the *VKORC1* gene − the locus-specific branch length (LSBL) test [16], the log of the ratio of heterozygosities (ln*RH*) test [17], and Tajima’s *D* [18] − using genome-wide data available for the West African, European and East Asian HapMap samples [19]. The tests yielded significant results in the East Asian sample. Interestingly, the rs9923231 SNP (g.-1639G>A), which was found to be a putative target of positive selection [14], is the main genetic determinant of AVK dose requirement and can alone explain between 25% to 30% of the dose variance among patients [4], [5], [6], [7]. This SNP, located in the promoter region, alters a *VKORC1* transcription factor binding site, leading to lower protein expression [20]. By decreasing VKORC1 activity, the derived -1639A allele thus confers an increased AVK sensitivity phenotype and patients carrying one and two -1639A alleles require on average respectively 25% and 50% lower daily warfarin doses than -1639G homozygous carriers to obtain the same anticoagulant effect [21], [22]. Understanding the processes of local adaption that may result in high levels of population differentiation and important interethnic differences in the required AVK dose is thus of particular relevance.

During these last few years, newer methods than those proposed by Ross *et al*. have been developed to detect the molecular footprints of positive selection. These methods are particularly well suited to detect classical signatures of selective sweeps, *i.e.* when a new advantageous mutation spreads rapidly to fixation in particular populations (the so-called ‘hard sweep’ model) [23]. Such a selective sweep occurs too quickly to leave enough time for recombination events to break down the linkage disequilibrium (LD), leading to a similar increase in frequency of alleles at nearby variants. Therefore, the pattern of genetic variation in the genomic region surrounding the selected allele may differ among populations [24], and the selected allele is expected to be carried by a long and frequent haplotype only in those populations that experienced the local adaptive event [25]. Signals of positive selection can thus be detected by looking for an increased genetic differentiation among populations (using methods such as *F_ST_* [26] and the Cross-Population Composite Likelihood Ratio (XP-CLR) test [24]), and an extended haplotype homozygosity (EHH) at the putatively selected locus (using methods such as the Cross-Population Extended Haplotype Homozygosity (XP-EHH) test [27] and the integrated Haplotype Score (iHS) [28]). These methods have proved to be powerful and largely complementary to detect and localize a selective sweep, and are more robust to ascertainment bias in SNP discovery than methods based on the allele frequency spectrum such as the Tajima’s *D* used by Ross *et al.* [14], [29].

In this study, we investigated whether and how positive selection has acted on the *VKORC1* gene locus using these complementary analytic methods. Our first objective was to determine (1) if the selective sweep is restricted to East Asia or if it is detected in other geographic regions, in particular Central South Asia and America, which are geographically close to East Asia, and (2) if it occurred in all East Asian populations or only in a few of them. Thus, we genotyped six *VKORC1* SNPs in the HGDP-CEPH Panel [30] which covers a much wider range of world populations – including 17 populations from East Asia – than the HapMap Panel in which positive selection at the *VKORC1* locus was initially evidenced. Furthermore, by expanding the analysis to a 2 Mb region encompassing the *VKORC1* gene, we sought to determine if the selective sweep identified around *VKORC1* was due to positive selection directly acting on this gene, or if it was caused by positive selection at a nearby linked gene resulting in genetic hitchhiking [23]. Finally, we discuss combining different methods for uncovering distinct selection signatures, in order to both increase power to detect a selective signal and precisely define its genomic location. We address the difficulty, even with such detailed analyses, in identifying the specific target of selection.

## Results

### VKORC1 Haplotype Study

**Figure 1:**
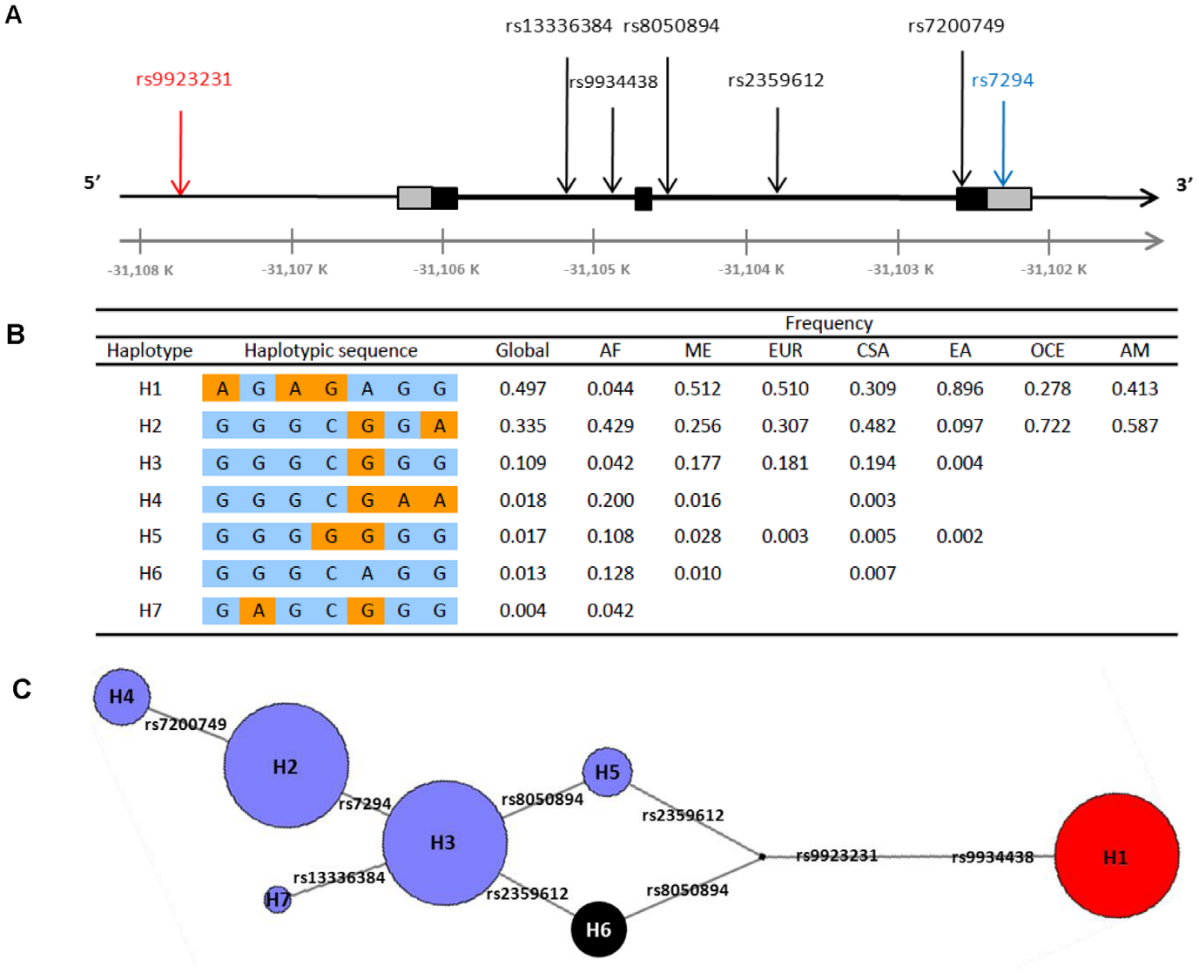
Results of the *VKORC1* haplotype study. (**A**) **Position of the seven SNPs along the** ***VKORC1*** **gene.** *VKORC1* is a 4.1 kb gene (GenBank accession number AY587020) located at 16p11.2. The three exons of the gene are represented as boxes, with 5′UTR and 3′UTR regions colored in grey and coding regions in black. Flanking and intronic regions are represented as thin and thick lines, respectively. The seven studied SNPs are shown in their sequential order along the *VKORC1* gene. The functional polymorphism rs9923231 located in the promoter, is highlighted in red and the SNP already present in the Illumina 650K chip in blue. Physical position along chromosome 16 is indicated in kb below. (**B**) **Distribution of** ***VKORC1*** **haplotypes at the global and regional level.** For each haplotype, SNPs are listed in the same sequential order than in Figure 1A. Ancestral and derived alleles are shown in blue and orange, respectively. Haplotype labels H1 to H7 were given according to the global haplotype frequency. AF, sub-Saharan Africa; ME, Middle East; EUR, Europe; CSA, Central South Asia; EA, East Asia; OCE, Oceania; AM, America. (**C**) **Median-joining network of the inferred** ***VKORC1*** **haplotypes at the global level.** Circles areas are proportional to the global haplotype frequency and branch lengths to the number of mutations separating haplotypes. Labels of haplotypes are indicated in corresponding circles, and labels of mutations on the network branches. The haplotype carrying the -1639A allele conferring the AVK sensitivity phenotype (H1) is shown in red and the ancestral haplotype (H6) in black.

A haplotype study of the 4.1 kb *VKORC1* gene was carried out with seven *VKORC1* SNPs genotyped in the 52 HGDP-CEPH population samples (Figure 1A). Haplotypes were reconstructed from these SNPs. Seven of these haplotypes had a frequency above 1% in at least one geographic region and were labeled H1 to H7 according to their frequency at the global level (Figure 1B). Four haplotypes are found in at least five geographic regions and only two are shared among all regions. The highest and lowest haplotype diversity values are observed in Sub-Saharan Africa (0.75±0.02) and East Asia (0.19±0.02), respectively. Most individuals carrying the ancestral haplotype (H6), *i.e.* the haplotype carrying the ancestral allele at each SNP, are from Sub-Saharan Africa (Figure 1B and Figure S1). Interestingly, the -1639A allele (rs9923231) conferring the increased sensitivity to AVK is carried by a unique haplotype (H1). This haplotype associated with AVK sensitivity is the most frequent at the worldwide level (49.7%) and shows an extremely high differentiation among geographic regions (Figure 1B). While rare in Sub-Saharan Africa (4.4%), it is found at intermediate frequencies in the Middle East, Europe, Central South Asia, Oceania and America (from 27.8% to 51.2%), and is largely predominant in East Asia (89.6%). The prevalence of H1 tends to be high in all of the 17 East Asian population samples investigated, ranging from 75% in She to 100% in Oroqen (Figure S1). However, the sample size is small for most of them, with 10 or less individuals.

The median-joining haplotype network describes the mutational relationships between the different *VKORC1* haplotypes inferred (Figure 1C). Haplotype H1 differs from the others by two nucleotide substitutions at the functional rs9923231 SNP and at the rs9934438 SNP, which are found in complete LD in all geographic regions (*D*’ = 1 and *r* ^2^ = 1, Figure S2).

### Detection of Signatures of Positive Selection

To support the hypothesis that positive selection has played a role in shaping patterns of genetic variation at *VKORC1*, four complementary methods were applied to detect signatures of selective sweeps in the genome. *F_ST_* and XP-CLR are both based on allele frequency differentiation, whereas XP-EHH and iHS are based on haplotype structure. Scores for the four test statistics were computed at both the regional and population levels for the seven *VKORC1* SNPs and for some other available SNPs [15] representing the expected neutral genomic background. For each score, a *p*-value was derived from the empirical distribution obtained from the genomic background (*cf*. Material and Methods). We considered as significant any *p*-value below 0.05. The results of the four tests are presented in Table 1 and Table 2.

**Table 1:** Results of the inter-regional *F_ST_*, intra-regional *F_ST_*, XP-EHH and iHS tests in the seven geographic regions.

Region	SNP	DAF^a^	Inter-regional *F_ST_* b	Inter-regional *F_ST_ p*-value^c^	Intra-regional *F_ST_* d	Intra- regional *F_ST_ p*-value^c^	XP-EHH score	XP-EHH *p*-value^e^	iHS score	iHS *p*-value^e^
Africa	rs7294	0.63	0.18	0.215	0.13	0.074	−0.94	0.833	−1.24	0.183
	rs7200749	0.20	0.48	0.217	0.02	0.643	−1.50	0.923	−0.31	0.748
	rs2359612	0.82	0.23	0.173	0.09	0.123	−1.15	0.875	−0.96	0.305
	rs8050894	0.16	0.25	0.143	0.09	0.125	−1.14	0.872	0.11	0.909
	rs9934438	0.04	0.36	0.029 *	0.10	0.058	−1.05	0.855	−0.03	0.974
	rs13336384	0.04	0.16	0.329	0.02	0.448	−1.06	0.858	0.14	0.883
	rs9923231	0.04	0.36	0.029 *	0.10	0.058	−1.01	0.845	−0.01	0.989
Middle East	rs7294	0.27	0.02	0.411	0.00	0.906	0.94	0.171	1.55	0.103
	rs7200749	0.02	0.00	0.670	0.02	0.310	1.58	0.069	0.65	0.492
	rs2359612	0.48	0.00	1.000	0.006	0.595	1.17	0.127	2.69	0.009 **
	rs8050894	0.54	0.00	0.849	0.002	0.667	1.15	0.132	−1.40	0.141
	rs9934438	0.51	0.00	0.946	0.01	0.481	1.05	0.149	−1.76	0.066
	rs13336384	0.00	0.005	0.044 *	0.00	1.000	1.07	0.145	NA	NA
	rs9923231	0.51	0.00	0.946	0.01	0.481	1.01	0.156	−1.76	0.066
Europe	rs7294	0.30	0.005	0.670	0.004	0.570	0.94	0.167	0.67	0.474
	rs7200749	0.00	0.02	0.477	0.00	1.000	1.50	0.077	NA	NA
	rs2359612	0.49	0.00	1.000	0.02	0.304	1.15	0.125	2.00	0.039 *
	rs8050894	0.51	0.00	1.000	0.02	0.286	1.14	0.128	−0.98	0.298
	rs9934438	0.51	0.00	0.993	0.02	0.304	1.05	0.145	−1.02	0.281
	rs13336384	0.00	0.005	0.071	0.00	1.000	1.06	0.142	NA	NA
	rs9923231	0.51	0.00	0.993	0.02	0.304	1.01	0.155	−1.02	0.281
Central South Asia	rs7294	0.49	0.06	0.042 *	0.07	0.026*	0.62	0.260	−0.54	0.550
	rs7200749	0.003	0.02	0.261	0.02	0.041*	1.25	0.116	NA	NA
	rs2359612	0.69	0.12	0.002 **	0.07	0.025*	0.89	0.187	1.00	0.280
	rs8050894	0.32	0.12	0.003 **	0.08	0.015*	0.87	0.191	−0.13	0.893
	rs9934438	0.31	0.10	0.006 **	0.07	0.020*	0.78	0.215	−0.20	0.834
	rs13336384	0.00	0.005	0.088	0.00	1.000	0.79	0.210	NA	NA
	rs9923231	0.31	0.10	0.006 **	0.07	0.020*	0.73	0.227	−0.20	0.834
East Asia	rs7294	0.10	0.21	0.063	0.02	0.311	2.68	0.011 *	1.99	0.040 *
	rs7200749	0.00	0.02	0.576	0.00	1.000	3.10	0.005 **	NA	NA
	rs2359612	0.10	0.39	0.005 **	0.02	0.317	2.89	0.008 **	1.92	0.047 *
	rs8050894	0.90	0.38	0.005 **	0.02	0.331	2.88	0.008 **	−1.20	0.200
	rs9934438	0.90	0.41	0.003 **	0.02	0.300	2.81	0.009 **	−1.27	0.174
	rs13336384	0.00	0.005	0.252	0.00	1.000	2.81	0.009 **	NA	NA
	rs9923231	0.90	0.41	0.003 **	0.02	0.300	2.773	0.010 *	−1.274	0.174
Oceania	rs7294	0.72	0.23	0.090	0.00	0.771	0.03	0.456	0.05	0.961
	rs7200749	0.00	0.005	0.401	0.00	1.000	0.51	0.285	NA	NA
	rs2359612	0.72	0.09	0.404	0.00	0.771	0.32	0.346	0.05	0.961
	rs8050894	0.25	0.12	0.327	0.02	0.530	0.29	0.355	0.50	0.584
	rs9934438	0.28	0.08	0.438	0.00	0.749	0.20	0.388	0.50	0.584
	rs13336384	0.00	0.009	0.014 *	0.00	1.000	0.21	0.384	NA	NA
	rs9923231	0.28	0.08	0.438	0.00	0.749	0.16	0.404	0.50	0.584
America	rs7294	0.58	0.11	0.320	0.17	0.195	0.76	0.207	NA	NA
	rs7200749	0.00	0.01	0.553	0.00	1.000	1.15	0.125	NA	NA
	rs2359612	0.59	0.02	0.674	0.17	0.190	0.96	0.162	NA	NA
	rs8050894	0.41	0.02	0.667	0.17	0.190	0.95	0.163	NA	NA
	rs9934438	0.41	0.01	0.743	0.17	0.190	0.88	0.178	NA	NA
	rs13336384	0.00	0.006	0.013 *	0.00	1.000	0.89	0.176	NA	NA
	rs9923231	0.41	0.01	0.743	0.17	0.190	0.85	0.185	NA	NA

a Derived allele frequency estimated at the global level.

b *F_ST_* estimated at the inter-regional level, *i.e.* between a given geographic region and the remaining ones.

c *P*-values are derived from the genome-wide empirical distribution of *F_ST_* values.

d *F_ST_* estimated at the intra-regional level, *i.e.* among populations within a region.

e *P*-values are derived from the empirical distribution of the iHS and XP-EHH scores along the chromosome 16.

* *p*<0.05; ** *p*<0.01; *** *p*<0.005.

NA: Not Applicable (for iHS: when a gap>200 kb between successive SNPs is found in the region in the region delimited by the SNPs where the EHH value drops below 0.05 around the core SNP).

**Table 2:** Results of the XP-CLR test in a 16 kb region centered on *VKORC1* in the seven geographic regions.

Region	Physicalposition	XP-CLRscore	XP-CLR*p-v*alue^a^
Africa	31005354	0.00	1.000
	31009354	0.00	1.000
	31013354	0.96	0.289
	31017354	0.58	0.348
	31021354	0.09	0.470
Middle East	31005354	4.00	0.138
	31009354	0.85	0.306
	31013354	3.28	0.158
	31017354	0.27	0.403
	31021354	6.25	0.092
Europe	31005354	0.54	0.351
	31009354	0.00	1.000
	31013354	2.63	0.186
	31017354	0.15	0.427
	31021354	2.40	0.198
Central South Asia	31005354	0.03	0.464
	31009354	0.00	1.000
	31013354	0.00	1.000
	31017354	0.01	0.476
	31021354	0.00	0.490
East Asia	31005354	24.08	0.032 *
	31009354	16.53	0.050 *
	31013354	30.49	0.023 *
	31017354	26.82	0.028 *
	31021354	43.44	0.012 *
Oceania	31005263	0.00	1.000
	31009263	0.00	1.000
	31013263	0.00	1.000
	31017263	0.00	1.000
	31021263	0.00	1.000
America	31005354	0.00	1.000
	31009354	0.00	1.000
	31013354	0.00	1.000
	31017354	0.01	0.587
	31021354	0.00	0.597

a *P*-values are derived from the empirical distribution of the XP-CLR scores along the chromosome 16.

* *p*<0.05; ** *p*<0.01; *** *p*<0.005.

**Figure 2:**
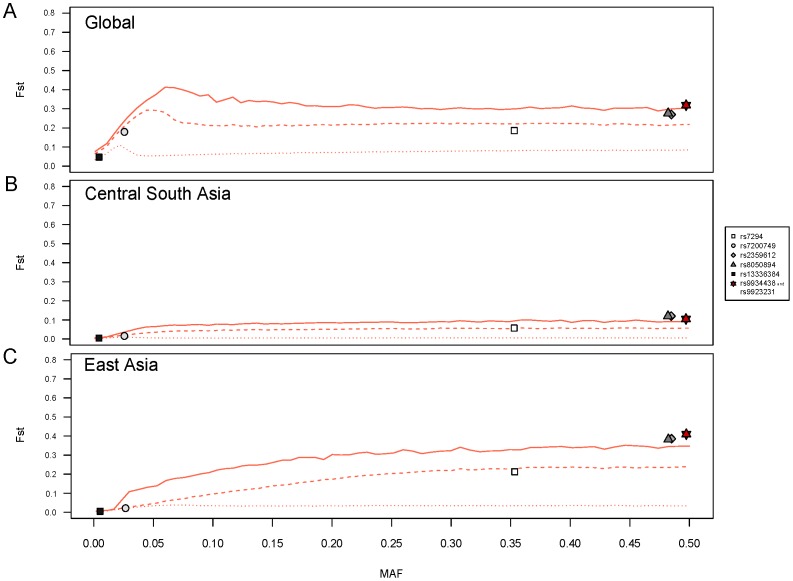
Atypical patterns of genetic differentiation observed for *VKORC1* SNPs. Genome-wide empirical distributions of *F_ST_* values were constructed from 644,143 SNPs having a MAF ≥0.001 at the global level. Individual values of *F_ST_* calculated for each of the seven *VKORC1* SNPs are plotted against their global MAF. The functional rs9923231 SNP is shown in red. The 50^th^, 95^th^ and 99^th^ percentiles are indicated as dotted, dashed and full red lines, respectively.

At the global level, when we evaluated the level of genetic differentiation among the seven HGDP-CEPH Panel geographic regions, an atypical pattern of genetic differentiation was detected for four *VKORC1* SNPs: rs2359612, rs8050894, rs9934438 and rs9923231 (*p*<0.05). The functional rs9923231 polymorphism and the rs9934438 SNP, in complete LD with each other, displayed *F_ST_* values falling above the 99^th^ percentile of the empirical genome-wide distribution (*F_ST_* = 0.32, *p* = 0.008) (Figure 2A). When global *F_ST_* values were computed among the 52 world populations, very similar results were obtained (Table S1). At the inter-regional level, *i.e.* between a given geographic region and the remaining ones, the same four *VKORC1* SNPs showed highly significant *F_ST_* values (*p*<0.01) when comparing Central South Asia and East Asia to the rest of the world (Table 1, Figures 2B and 2C). Regarding East Asia, the highest *F_ST_* values (*F_ST_* = 0.41, *p* = 0.003) were also observed for the two SNPs, rs9923231 and rs9934438. For the other geographic regions, no *VKORC1* SNP displayed an inter-regional *F_ST_* value as much significant as the ones observed for Central South Asia and East Asia (Table 1 and Figure S3). At the intra-regional level, *i.e.* among populations within a region, no extreme pattern of genetic differentiation (*p*<0.01) was observed for any *VKORC1* SNP in any geographic region (Table 1 and Figure S4).

The XP-CLR test applied to each geographic region also provided evidence of an atypical pattern of genetic differentiation at the *VKORC1* gene locus, with XP-CLR scores in East Asia ranging from 16.53 (*p* = 0.050) to 43.44 (*p* = 0.012) in the 16 kb genomic region centered on *VKORC1* (Table 2). For each of the other six geographic regions, the XP-CLR scores were very low, supporting the existence of a selective sweep restricted to East Asia. In this geographic region, when the XP-CLR test was performed for each population, all of the 17 population samples, except Oroqen, showed this extreme pattern of genetic differentiation, with at least three significant XP-CLR scores out of the five scores computed in the 16 kb genomic region surrounding *VKORC1* (Table S2). As most of the SNPs in the *VKORC1* genomic region have reached fixation in the Oroqen sample, XP-CLR scores could be calculated for only very few SNPs on either side of *VKORC1*, making difficult the interpretation of XP-CLR results in this sample.

Regional results obtained with the extended haplotype-based XP-EHH test indicated that the unusual pattern of genetic differentiation observed at the *VKORC1* gene locus resulted from a selective sweep in East Asia. Significant XP-EHH scores, ranging from 2.68 (*p* = 0.011) to 3.10 (*p* = 0.005), were observed for the seven *VKORC1* SNPs in East Asia, while no significant values were observed for any other geographic region (Table 1). For East Asian populations, evidence for a selective sweep was detected in all 17 population samples with significant XP-EHH scores for each of the seven *VKORC1* SNPs, ranging from 1.84 (*p* = 0. 049) in the Dai sample for rs7294, to 3.78 (*p* = 0.004) in the Tujia sample for rs8050894 (Table S3).

With the iHS test, only two *VKORC1* SNPs (rs7294 and rs2359612) exhibited significant iHS scores in East Asia (*p = *0.040 and 0.047, respectively; Table 1). Two other significant scores were observed for the rs2359612 SNP in the Middle East (2.69, *p = *0.009) and Europe (2.00, *p* = 0.039). At the population level in East Asia, only three samples (Hezhen, Lahu, and Yakut) displayed significant iHS scores for two, three and four SNPs, respectively (Table S3).

**Figure 3:**
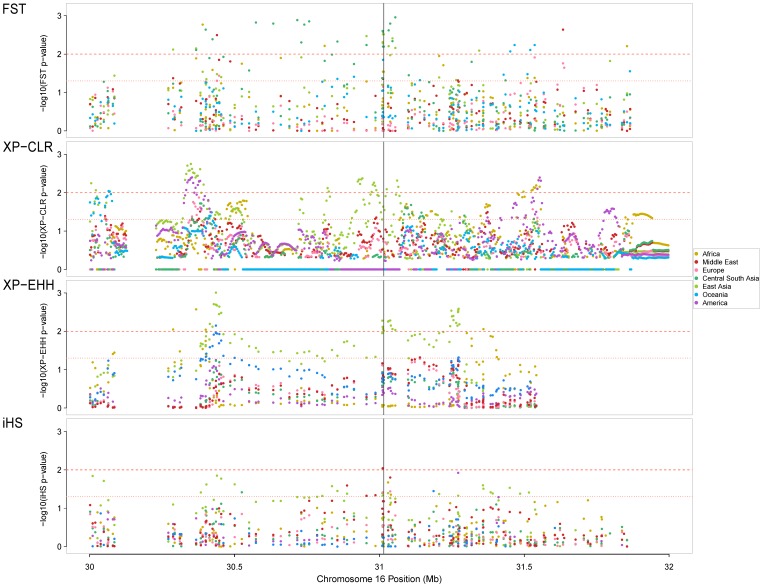
Distribution of –log_10_ (*p-*values) for four selection tests across a 2 Mb region centered on *VKORC1*. A black vertical line indicates the physical position of *VKORC1* on chromosome 16. Horizontal red dotted and dashed lines show 0.05 and 0.01 chromosome-wide significance levels, respectively. The selection tests (inter-regional *F_ST_*, XP-CLR, XP-EHH and iHS, respectively) were separately applied in each of the seven geographic regions.

**Figure 4:**
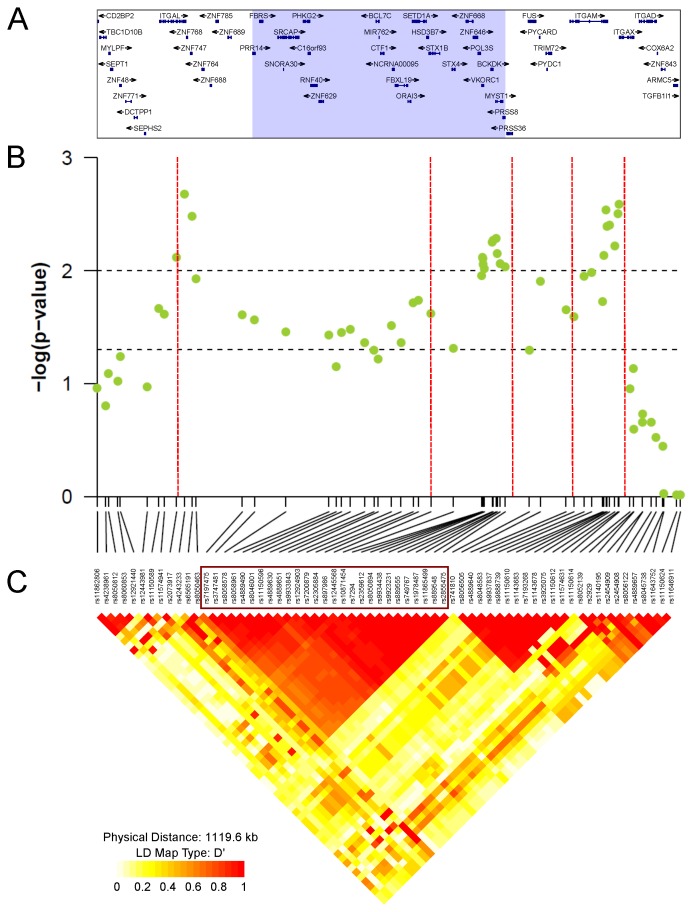
Detailed analysis of a 1.1 Mb genomic region surrounding the *VKORC1* gene locus in East Asia. The boundaries of the region displayed (chr16:30,271,572-31,391,123; UCSC human genome build hg18) were chosen so as to include the three clusters of significant scores detected in East Asia by the selection tests in the 2 Mb region centered on *VKORC1* (Figure 3). (**A**) **Name and location of genes.** Exons are displayed as blue boxes and the transcribed strand is indicated with an arrow. Genes located in the block of strong LD encompassing *VKORC1* and including the SNPs in the red box shown in Figure 4C, are highlighted in the grey area. (**B**) **XP-EHH results in East Asia.** The significance of the XP-EHH scores (−log_10_ empirical *p*-value) are shown for individual SNPs with a MAF ≥0.01 in East Asia. Horizontal dashed lines indicate 0.05 and 0.01 chromosome-wide significance levels. Recombination hotspots detected in HapMap Phase II data are indicated by red vertical dotted lines. The data and methods used to derive these hotspots are available from the HapMap website (http://www.hapmap.org/) [83], [84]. (**C**) **LD plot.** Pairwise LD values, depicted as *D*’, are shown for SNPs with a MAF ≥0.01 in East Asia. *D*’ values are displayed in different colors from yellow to red for *D*’ = 0 to *D*’ = 1, respectively. The red box highlights SNPs included in the LD block encompassing *VKORC1.* The plot was produced using the snp.plotter R package [74].

The four selection tests consistently evidenced the signature of a selective sweep involving the *VKORC1* genomic region in East Asia. However, this result did not allow us to determine with certainty that *VKORC1* is the direct target of positive selection. A linked gene could be the target instead, resulting in genetic hitchhiking of *VKORC1* [23]. In an attempt to seek the true target of positive selection, we probed the downloaded chromosome 16 genotypes [15] with the four tests for selection and examined the results over an extended 2 Mb genomic region centered on *VKORC1*. We focused on clusters of selection test scores with highly significant *p*-values (*p*<0.01) for East Asia only. Three clusters were observed (Figure 3): (i) ∼ 570 kb downstream of *VKORC1*, the first cluster was found with partially overlapping clusters of extreme XP-CLR and XP-EHH scores over a region of 64 and 39 kb, respectively, involving the genes *ITGAL*, *ZNF768*, and *ZNF747*; (ii) at or close to *VKORC1* genomic position, the second cluster was determined by overlapping clusters of extreme *F_ST_* values when comparing East Asia to the rest of the world (with the lowest *p*-values observed for the same two *VKORC1* SNPs evidenced before, rs9923231 and rs9934438) and extreme XP-CLR and XP-EHH scores. These clusters ranged in size from 45 to 244 kb; (iii) ∼ 230 kb upstream of *VKORC1*, the third cluster of 32 kb was found with XP-EHH and concerned the genes *ITGAM* and *ITGAX*. If SNPs within clusters are in high LD (*D*’≥0.97, except for one SNP in the third cluster), only limited LD exists between the SNPs located in the different clusters (Figure 4 and Figure S5) and several recombination hotspots are present between these clusters (Figure 4). This suggests that each of the three clusters represents a different adaptive event.

Examination of the second cluster showed that *VKORC1* is contained in a block of strong LD spanning ∼ 505 kb in East Asia (Figure 4 and Figure S5). Similar LD blocks were observed for Central South Asia and Europe, and to a lesser extent, for the Middle East (Figure S5). This LD block encompasses 25 genes (Figure 4). We used the most extreme *F_ST_*, XP-CLR and XP-EHH scores in order to spatially localize a target of selection within the LD block. Significant XP-CLR scores (*p*<0.05) were found in a 350 kb region encompassing 19 genes including *VKORC1* (Table S4). XP-EHH scores were almost all significant at the 0.05 threshold but four adjacent genes *VKORC1*, *BCKDK*, *MYST1* (*KAT8*) and *PRSS8* displayed most extreme XP-EHH scores (*p*<0.01). Clusters of highly significant *F_ST_* values when comparing East Asia to the rest of the world (*p*<0.01) and significant global *F_ST_* values (*p*<0.05) were also found for these four genes (Table S5). It is thus probable that the selective pressure has targeted one of these genes.

### When did the -1639A VKORC1 Allele begin to Increase in East Asia?

The time at which the frequency of the -1639A allele started to increase in East Asia was estimated by using a maximum-likelihood method [31] with the 17 East Asian HGDP-CEPH sample data. Our analysis yielded an age estimate of 181 generations (95% CI: 128–256 generations). Assuming a generation time of 25 years, the expansion therefore occurred about 4,525 years ago (95% CI: 3,200–6,400 years).

## Discussion

Numerous genes involved in absorption, distribution, metabolism and excretion (ADME) of drugs, exhibit evidence of recent positive selection and/or high population differentiation levels [32]. However, there are fewer examples of the action of natural selection on genes involved in the pharmacodynamics of drugs, such as *VKORC1*. Although numerous surveys have examined the genetic polymorphism of *VKORC1* in samples from diverse ethnic origins [13], [20], [33], [34], [35], [36], [37], these studies provided an incomplete picture of haplotype diversity because different sets of SNPs were used and worldwide coverage was incomplete. In this study, we took advantage of the worldwide coverage of the HGDP-CEPH Panel to provide the first detailed analysis of *VKORC1* population diversity using the same set of SNPs. Haplotype analysis revealed that the -1639A derived allele that confers AVK sensitivity is carried by a unique haplotype in all 52 population samples investigated. This haplotype associated with AVK sensitivity is predominant in East Asia, rare in Sub-Saharan Africa and occurs at intermediate frequencies in other geographic regions. Because it is found in Sub-Saharan Africa and other world populations, this haplotype is probably rather old. Its geographic distribution leads to striking differences between East Asian and non East Asian samples for genetic susceptibility to AVK sensitivity.

One explanation for worldwide diversity of this haplotype could be positive selection. This hypothesis was supported by five genome-wide scans that found atypical patterns of the allele frequency spectrum [38], extended LD [39], [40], and unusual genetic differentiation [40], [41], [42] in a 450 kb genomic region encompassing *VKORC1*. When specified, the target population was Asian [38], [40]. Ross *et al.* [14] found evidence of positive selection at *VKORC1* in the East Asian HapMap sample, based on the level of genetic diversity (ln*RH* test [17]), genetic differentiation (LSBL test [16]) and allele frequency spectrum (Tajima’s *D* [18]).

In this study, we provided compelling evidence of positive selection at the *VKORC1* gene locus in East Asia and only in this geographic region. A footprint of natural selection was found in each of the widely distributed 17 HGDP-CEPH East Asian population samples. By using four different tests of positive selection and by assessing significance at a given locus on the basis of an empirical distribution derived from the genomic background, we believe we can be confident that positive selection, rather than demographic forces, accounts for the data presented here. Indeed, it is well known that large allele frequency differences between populations are not infallible proofs of positive selection: these can also result from genetic drift, migration and other neutral demographic processes [43], [44]. This might be the explanation for the significant inter-regional *F_ST_* values observed in Central South Asia (Table 1 and Figure 2B).

Because the XP-EHH test is designed to detect fixation events that are relatively young (∼ 30,000 years) [27], the selective event we have detected is likely to be rather recent. This is indeed supported by an age estimate of 4,525 years (95% CI: 3,200–6,400 years) for the time at which the *VKORC1* -1639A allele started to increase in frequency in East Asia. The poor performance of the iHS test that detected only very few signals of positive selection in this study could have been predicted since its power to detect selective sweeps involving alleles near fixation is known to be low [28], [45]. By contrast, XP-EHH and XP-CLR perform better when the allele targeted by selection is near fixation and indeed showed strong evidence of a selective sweep in this study [24], [27].

In an attempt to determine if the *VKORC1* gene has been the direct target of positive selection or if it reflects genetic hitchhiking [23], we extended our analysis to a 2 Mb region surrounding the *VKORC1* gene (Figure 3). Apart from the highly significant footprint of positive selection localized in the *VKORC1* region, two other significant signals, at ∼ 570 kb downstream and ∼ 230 kb upstream of *VKORC1*, were detected with XP-CLR and/or XP-EHH in East Asia. These two regions contains genes that belong to the same integrin family – specifically to the CD11 gene cluster: *ITGAL* downstream, and adjoining genes *ITGAM* and *ITGAX* upstream – involved in immune functions and being thus good candidates for positive selection [46], [47], [48]. However, since SNPs located in these integrin genes show limited LD with those of *VKORC1*, a single adaptive event is unlikely. Apart from East Asia, the *ITGAL* region showed signals of positive selection in other geographic regions (America with XP-CLR, and Sub-Saharan Africa and Oceania with XP-EHH), arguing for a different evolutionary history from that of *VKORC1*, which was only found in East Asia. This observation emphasizes the need for studying the geographic distribution of a selective event in a wide range of genetically diverse populations, as per Scheinfeldt *et al.* [49] who, after performing a detailed analysis of a 3 Mb region surrounding a gene showing strong footprints of positive selection, discovered patterns of genetic variation consistent with the presence of a cluster of three independent selective events occurring in different populations. By extending their analysis to the entire genome, they identified several other genomic regions exhibiting evidence for the presence of multiple and independent selective targets, suggesting that clusters of adaptive evolution, such as the one detected herein, are widespread in the human genome.

After delimitating the selective signal for *VKORC1* by analyzing selective events identified in the 2 Mb region just described, we aimed at precisely mapping the gene targeted by positive selection. *VKORC1* is located in a ∼ 505 kb LD block in East Asia containing 25 genes (Figure 4), and the selective pressure could have targeted any gene in this LD block. We used *F_ST_*, XP-CLR and XP-EHH scores to spatially localize possible targets of positive selection within the LD region. A block of four adjacent genes –*VKORC1, BCKDK*, *MYST1,* and *PRSS8*– was found to be the most likely selective target (Table S4).

*BCKDK* codes for the mitochondrial branched chain ketoacid dehydrogenase kinase. *MYST1* and *PRSS8* are two immunity-related genes, listed as candidates for positive selection in several databases [40], [42], [50]. If, indeed, one of these three genes is the target of the selective sweep detected here, it should contain a functional variant of high frequency in East Asia and we did not find such a variant in HapMap data.

Assuming that selection has directly targeted the *VKORC1* gene, the advantage would then probably be related to vitamin K metabolism,vitamin K being the only known substrate of VKORC1. This vitamin plays a crucial role in the synthesis of vitamin K-dependent (VKD) proteins, especially blood coagulation factors, which requires VKORC1 activity [51], [52]. Large geographic differences in dietary vitamin K intake, especially in vitamin K2, exist between human populations, with the highest plasma levels found in Asian populations, as compared to Europeans and Africans [53], [54]. These differences could be explained by the wide consumption of fermented soybean food (*natto*) - a major source of vitamin K2 - in East Asia [55], [56]. It is then possible that, at some points in the history of East Asian populations, these high levels of vitamin K intakecould have been deleterious and created a selective pressure against *VKORC1* gene expression and coagulant activity. There is, however, no report so far of a deleterious effect associated with a high consumption of vitamin K and it is more the low dietary vitamin K intake that is problematic, hampering the adequate synthesis of VKD proteins in extrahepatic tissues notably bone and arterial vessels [57]. An alternative hypothesis could be that a naturally occurring environmental molecule of AVK type - such as a coumarin derivative - specifically found in East Asia, exerted a selective pressure on the *VKORC1* gene in populations of this region during their recent history. Such molecules are present in the nature, as illustrated by the example of the sweet clover disease that affected cattle in Canada and North America in the 1920’s. Sweet clover hay, used to feed cattle, contains a natural coumarin that is oxidized in mouldy hay to form dicoumarol, a hemorrhagic agent. Its discovery led to the synthesis of coumarin derivatives used in clinical application as oral anticoagulants since the 1940’s [58], [59]. Evidence of an effect of warfarin in shaping *VKORC1* genetic diversity could be found in rats and mices. Indeed, since the introduction in the 1950’s of this molecule as rodenticide, mutations in the *VKORC1* gene conferring warfarin resistance have spread in rodent populations but the mechanisms by which they lead to warfarin resistance are still not elucidated [60], [61], [62], [63].

In conclusion, we found that the *VKORC1* genomic region exhibits diversity patterns consistent with the action of positive selection in East Asia. Nearly complete selective sweeps, such as the one described herein, are believed to be rare in recent human adaptive history [64], [65], [66], [67]. This selective event is probably responsible for the spread of the derived -1639A allele conferring the increased AVK-sensitive phenotype in East Asian populations and contributes to present-day differences among human populations in the genetic sensitivity to AVK. A detailed analysis of the extended *VKORC1* genomic region revealed selective signals at several independent genetic loci, indicating a complex evolutionary history for this chromosome 16 region. Our evolutionary analysis emphasizes the importance of considering the surrounding genomic region of a candidate gene for selection in order to avoid erroneous conclusions about the true target of selection. We show here that the gene targeted by selection could be either *VKORC1* or another gene located in the 45 kb region covered by selective sweep detected in East Asia. Our ability to identify the target of selection may be limited by the number of genetic polymorphisms investigated. Examining the selective signal with more genetic variation using whole-genome sequences from the 1000 Genomes Project [68] may well improve the mapping of the gene targeted by selection. Furthermore, allele frequency spectrum bias tends to be minimized with whole genome sequences, which may allow the use of tests for natural selection based on this spectrum.

## Materials and Methods

### The HGDP-CEPH Panel

We used the HGDP-CEPH Panel that presently includes 1,064 individuals from 52 populations worldwide [69]. For the analysis presented here, the standardized subset panel H952 containing no first nor second degree relative pairs, was used [70]. This subpanel includes 952 individuals grouped into seven broad geographic regions as defined by Li *et al.* [15]: Sub-Saharan Africa (N = 105), the Middle East and Mozabites from north Africa (N = 163), Europe (N = 158), Central South Asia (N = 202), East Asia (N = 232), Oceania (N = 28) and America (N = 64). A full description of the 52 samples included in the HGDP-CEPH Panel is provided in Table S6.

### SNP Genotyping

A total of 940 individuals from the original H952 subpanel were previously genotyped by Li *et al.* [15] with the Illumina HumanHap 650 K platform and their genotypes at 644,258 autosomal SNPs were downloaded from the public HGDP-CEPH database (http://www.cephb.fr/en/hgdp/). Only one SNP (rs7294) from this dataset is located in the *VKORC1* gene. We additionally genotyped six SNPs in *VKORC1* in the 940 individuals, using the TaqMan® SNP Genotyping Assay-by-Design method in 5 µl reaction volumes according to the manufacturer's protocol (Applied Biosystems, Foster City, CA): rs9923231 (g.-1639G>A) located in the promoter region, rs13336384 and rs9934438 in the first intron, rs2359612 and rs8050894 in the second intron, and rs7200749 in the third exon (Figure 1A). Missing genotype rates varied from 0.5% to 2.2% for SNPs rs7200749 and rs9934438, respectively. Since the two SNPs rs9923231 and rs9934438 were found in complete LD in the seven geographic regions (Figure S2), we were able to impute the missing genotypes of a given SNP using available information from the other, leading to a total of 0.96% missing genotypes for these two SNPs. No significant deviations from the Hardy-Weinberg proportions were observed for any *VKORC1* SNP in any of the 52 population samples at the 0.01 significance level (data not shown). Allele frequency distributions of the seven *VKORC1* SNPs in the 52 population samples are shown in Figure S6.

### Statistical Analysis

#### *VKORC1* haplotype study

To investigate the worldwide diversity of the *VKORC1* gene, we conducted a haplotype study using the seven genotyped SNPs. A total of 931 individuals with less than three missing genotypes were included in the haplotype reconstruction. For each geographic region, haplotype frequencies were estimated with the Bayesian statistical method implemented in Phase v2.1 [71] using defaults parameters. To avoid the convergence of the algorithm to a local maximum, we ran it 10 times with different random seeds and kept the output from the run with the best average value. The worldwide haplotype frequencies were then calculated as the weighted average of the frequencies estimated in each of the seven geographic regions. Similar results were obtained when a single pooled sample of all individuals was considered in the haplotype frequency estimation (data not shown). Since information on ancestral allele state is required to distinguish between ancestral and derived haplotypes, we used the snp131OrthoPt2Pa2Rm2.txt file downloaded from the UCSC genome browser (http://genome.ucsc.edu/) which provides the orthologous alleles in chimpanzee, orangutan and rhesus macaque. For each SNP, the allele shared by the three species was identified as the ancestral allele. Haplotype networks were drawn with the Network v4.5.1.6 software (http://www.fluxus-engineering.com/), using the median-joining algorithm which builds the minimum spanning network from the given haplotypes by favoring short connections [72]. LD analyses were performed with Haploview v4.1 [73] and the snp.plotter R package [74], using Lewontin’s disequilibrium coefficient *D*’ [75] and the correlation coefficient *r* ^2^ [76].

### Detection of Signatures of Positive Selection

To explore whether *VKORC1* has evolved under positive selection in humans, we looked for two distinct genetic patterns of a selective sweep that are expected to remain detectable in the genome over different time scales after the action of natural selection: (i) an important genetic differentiation among populations nearby the locus of interest, and (ii) the presence of unusually frequent and long haplotypes in the surrounding genomic region. For each method, we used an outlier approach to calculate the *p*-values of the computed scores. Under this approach, an empirical distribution is constructed using other SNPs in the genome that are assumed to be neutral and to represent the genomic background under neutrality. An empirical *p*-value is computed that corresponds to the proportion of values from the empirical distribution that are higher than the value observed at the locus of interest. If the value obtained for the SNP of interest is greater than the 95^th^ percentile (*p*<0.05) of the empirical distribution, positive selection is invoked. For that purpose, we used the empirical distributions obtained from the scores calculated either on a genome-wide (all autosomal chromosomes) or chromosome-wide (chromosome 16, where *VKORC1* is located) basis.

First, we used two statistics, *F_ST_* and XP-CLR, which measure the genetic differentiation among human populations [24], [26]. These methods are able to detect selective sweeps that have occurred up to 75,000 years ago [77]. The fixation index *F_ST_* [78] quantifies the proportion of genetic variance explained by allele frequency differences among populations. *F_ST_* ranges from 0 (for genetically identical populations) to 1 (for completely differentiated populations). We calculated *F_ST_* values using the BioPerl module PopGen [79] for each autosomal SNP with a minor allele frequency (MAF) ≥10^−3^ (644,143 SNPs) at three different levels: (i) global level (either among the seven HGDP-CEPH Panel geographic regions or among the 52 Panel populations), (ii) inter-regional level (each geographic region versus the remaining ones), and (iii) intra-regional level (among populations within a region). Since *F_ST_* strongly correlates with heterozygosity [41], [80], [81], empirical *p*-values were calculated within bins of 10,000 SNPs grouped according to MAF. The resulting distributions represent the average genetic differentiation of human populations corrected for heterozygosity.

We next applied the XP-CLR test [24] which identifies selective sweeps in a population by detecting significant genetic differentiation in an extended genomic region of interest as compared to a reference population. This method presents both the advantages of being robust to ascertainment bias and of not requiring any information on haplotypes, thus avoiding errors of haplotype estimation from genotype data. XP-CLR scores were computed at regularly spaced grid points (every 4 kb) across chromosome 16 using the genotypes from SNPs within overlapping windows of 0.1 cM around each grid point. To account for different SNP densities among genomic regions, we restricted to 200 the maximal number of SNPs used to compute a XP-CLR score within the 0.1 cM genomic region, by removing excess SNPs at random. We applied this method by considering all SNPs with a MAF ≥10^−3^ on chromosome 16 at both the regional and population levels (17,729 SNPs). *P*-values were calculated from the empirical distribution of the collected scores obtained with these SNPs. XP-CLR requires the definition of a reference population: the Sub-Saharan African samples were used as a reference for non Sub-Saharan African regions, and the European samples as a reference for Sub-Saharan Africa. For the analyses performed at the population level, we defined the Yoruba as the reference for non Sub-Saharan African samples, and the French for Sub-Saharan African samples.

The second class of methods that we used is based on EHH, *i.e.* the sharing of identical alleles across relatively long distances by most haplotypes in population samples [25]. In brief, the EHH is computed for a given SNP (the core SNP) of a sequence being interrogated for a selective sweep. In the absence of a selective sweep, recombination events break down haplotypes relatively rapidly with time and with increasing distance from the core SNP. In the case of a selective sweep, LD tends to maintain the haplotype carrying the selected allele, and the relative frequency of this (favored) haplotype will increase with time leading to so-called EHH. Integration of genetic distance in both directions from the core SNP can be used to discriminate between selected and non-selected alleles, and be applied to ancestral and derived alleles. Analytic methods based on EHH are able to detect recent selective sweeps (*i.e.* those occurring less than 30,000 years ago [77]). Such analyses require haplotype data. We used fastPHASE v1.3.0 EM algorithm [82] to infer haplotypes with chromosome 16 SNPs for individuals from each geographic region. For each region, the *K*-selection procedure was first run several times in order to define the optimal number of clusters of similar haplotypes by minimizing chance error rates. Ultimately, phase was determined with *K* = 6 for Oceania, *K* = 14 for Europe and Central-South Asia and *K* = 12 for the remaining regions. Using these values, the EM algorithm was then run with 20 random starts and 25 iterations.

Once haplotypes were reconstructed, we computed the XP-EHH statistic [27] that compares the integrated EHH computed in a test population versus that of a reference population. Therefore, this method detects a sweep in which the selected allele has risen to near fixation in one population but remains polymorphic in the other. XP-EHH scores were computed using the same parameters as those described in Sabeti *et al*. (2007). Reference populations were defined as for XP-CLR.

We finally applied the iHS [28] that compares the rate of EHH decay observed for both the derived and ancestral allele at the core SNP. An extremely positive or negative value at the core SNP provides evidence of positive selection with unusually long haplotypes carrying the ancestral or the derived allele, respectively. The raw iHS scores were computed using the iHS option implemented in the WHAMM software developed by Voight *et al.* (2006). The scores were standardized to have null mean and unit variance in 5% bins of the derived allele frequency at the core SNP. Information on ancestral allele state was obtained from the snp131OrthoPt2Pa2Rm2.txt file downloaded from the UCSC website. We were unable to determine with certainty the ancestral allele status of 111 SNPs on chromosome 16 and we removed them from the analysis.

XP-EHH and iHS scores were calculated for all available SNPs on chromosome 16 (19,733 and 19,622, respectively) at both the regional and population levels. The resulting distributions were used to calculate empirical *p*-values.

The genetic map used for applying XP-CLR, XP-EHH and iHS was retrieved from release 22, build 36 of HapMap (www.hapmap.org).

### Age of the Expansion of the -1639A *VKORC1* Allele in East Asia

We inferred the age at which the -1639A allele started to increase in frequency in East Asia by estimating the age of the most recent common ancestor carrying this allele in East Asia using the likelihood-based method implemented in the Estiage program [31]. This method assumes that all individuals derive from a common ancestor who introduced the mutation *n* generations ago. Estimation of *n* is based on the length of the haplotype shared by the individuals, which is estimated through the identification of recombination events on the ancestral haplotype by taking into account allele frequencies and recombination rates. We estimated *n* using only one haplotype per East Asian population sample (*i.e.*, 17 haplotypes). For each population, this one haplotype was constructed by taking at each locus over a 6 Mb region the allele the most frequently seen in individuals from the population carrying the -1639A allele. A mutation rate of 10^−6^ per individual and per generation, and a 25-year generation time were assumed.

## Supporting Information

Figure S1 **Distribution of** ***VKORC1*** **haplotypes in the 52 HGDP-CEPH samples.** The haplotype carrying the -1639A allele (H1) is represented in red and the ancestral haplotype (H6) in black.
(TIF)

Figure S2 **Pairwise LD between the seven** ***VKORC1*** **SNPs at the regional and global level.** Red squares indicate statistically significant (logarithm of odds >2) LD between the pair of SNPs, as measured by the *D*’ statistic [75] with the Haploview software [73]; darker colors of red indicate higher values of *D*’, up to a maximum of 1. White squares indicate pairwise *D*’ values of <1 with no statistically significant evidence of LD. Blue squares indicate pairwise *D*’ values of 1 but without statistical significance.
(TIF)

Figure S3 **Genome-wide empirical distributions of inter-regional** ***F_ST_*** **values against MAF in the seven geographic regions.** Empirical distributions of *F_ST_* were constructed by calculating an *F_ST_* value for 644,413 SNPs having a MAF ≥0.001 at the global level. Individual values of *F_ST_* calculated for each of the seven *VKORC1* SNPs are plotted against their global MAF. The functional rs9923231 SNP is shown in red. The 50^th^, 95^th^ and 99^th^ percentiles are indicated as dotted, dashed and full red lines, respectively.
(TIFF)

Figure S4 **Genome-wide empirical distributions of intra-regional** ***F_ST_*** **values against MAF in the seven geographic regions.** Empirical distributions of *F_ST_* were constructed by calculating an *F_ST_* value for all SNPs having a MAF ≥0.001 at the intra-regional level. Individual values of *F_ST_* calculated for each of the seven *VKORC1* SNPs are plotted against the regional MAF. The functional rs9923231 SNP is shown in red. The 50^th^, 95^th^ and 99^th^ percentiles are indicated as dotted, dashed and full red lines, respectively.
(TIFF)

Figure S5 **LD patterns over a 2 Mb region centered on** ***VKORC1*** **in the seven geographic regions.** Pairwise LD, depicted as *D*’, is shown for SNPs with a MAF ≥0.05 at the global level. *D*’ values are displayed in different colors from yellow to red for *D*’ = 0 to *D*’ = 1, respectively. The plot was produced using the snp.plotter R package [74]. The vertical dashed lines delineate *VKORC1* gene position.
(TIF)

Figure S6 **Allele frequency distribution of the seven** ***VKORC1*** **SNPs in the 52 HGDP-CEPH samples:** rs9923231 **(A)**, rs13336384, **(B)** rs9934438 **(C)**, rs8050894 **(D)**, rs2359612 **(E)**, rs7200749 **(F)** and rs7294 **(G)**. The derived and ancestral alleles are represented in orange and blue, respectively.
(TIF)

Table S1 Global *F_ST_* values among populations and among regions for the seven *VKORC1* SNPs.
(XLS)

Table S2 Results of the XP-CLR test in a 16 kb region centered on *VKORC1* in the 52 HGDP-CEPH samples.
(XLS)

Table S3 Results of the XP-EHH and iHS tests in the 52 HGDP-CEPH samples.
(XLS)

Table S4 Results of the XP-CLR test in the ∼ 500 kb genomic region of the LD block encompassing *VKORC1* in East Asia.
(XLS)

Table S5 Results of the XP-EHH, iHS tests, inter-regional *F_ST_* and global *F_ST_* for all SNPs located in the linkage disequilibrium block encompassing *VKORC1* in East Asia.
(XLS)

Table S6 Description of the 52 HGDP-CEPH samples grouped into seven main geographic regions.
(XLS)
