# Supplementary figures and images for: Positive Selection in the Chromosome 16 VKORC1 Genomic Region Has Contributed to the Variability of Anticoagulant Response in Humans
Source: PLoS One. 2012 Dec 28;7(12):e53049. doi: 10.1371/journal.pone.0053049 (PMC3532425; doi:10.1371/journal.pone.0053049)

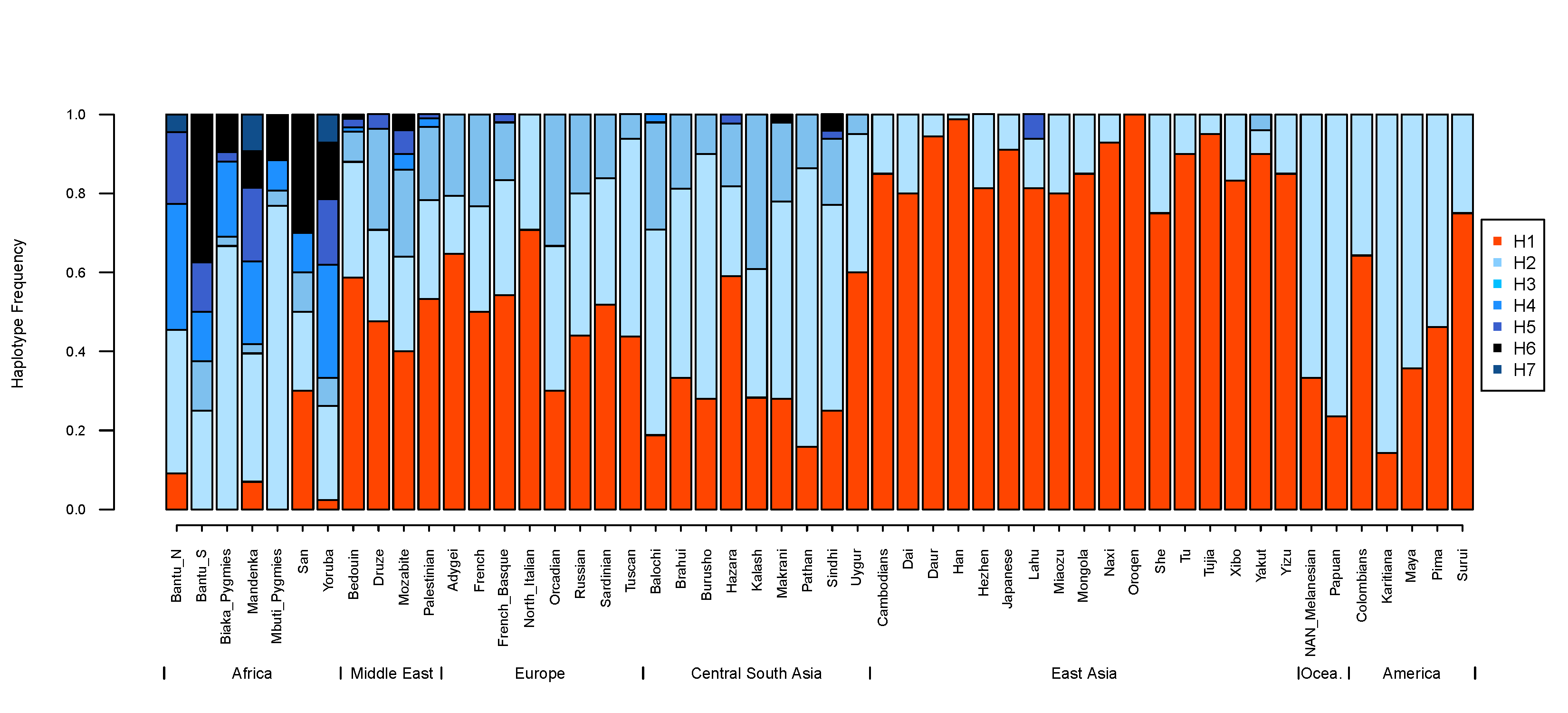

Supplement: Figure S1 — Distribution of VKORC1 haplotypes in the 52 HGDP-CEPH samples. The haplotype carrying the -1639A allele (H1) is represented in red and the ancestral haplotype (H6) in black. (TIF) [file pone.0053049.s001.tif]

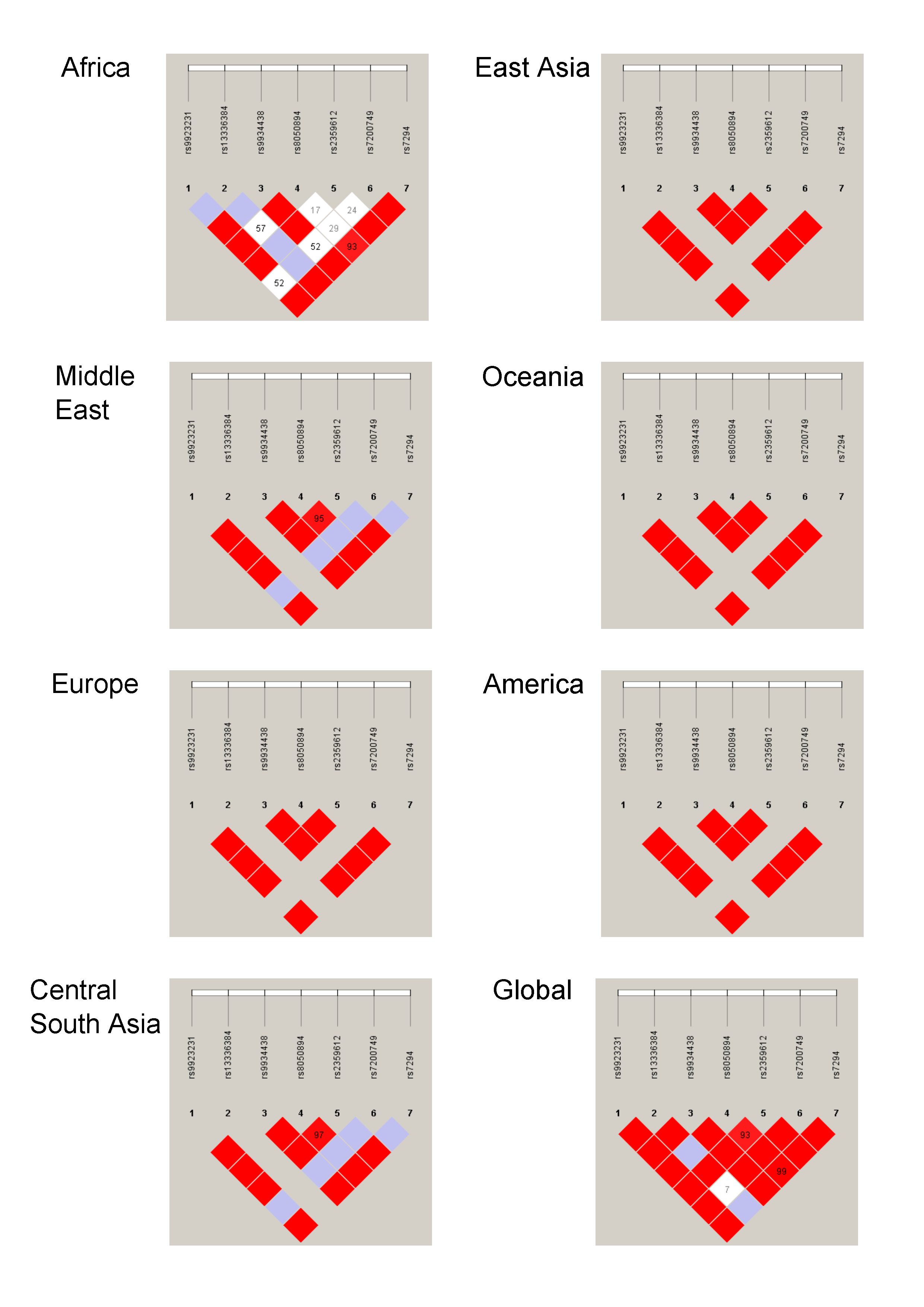

Supplement: Figure S2 — Pairwise LD between the seven VKORC1 SNPs at the regional and global level. Red squares indicate statistically significant (logarithm of odds >2) LD between the pair of SNPs, as measured by the D’ statistic [75] with the Haploview software [73]; darker colors of red indicate higher values of D’, up to a maximum of 1. White squares indicate pairwise D’ values of <1 with no statistically significant evidence of LD. Blue squares indicate pairwise D’ values of 1 but without statistical significance. (TIF) [file pone.0053049.s002.tif]

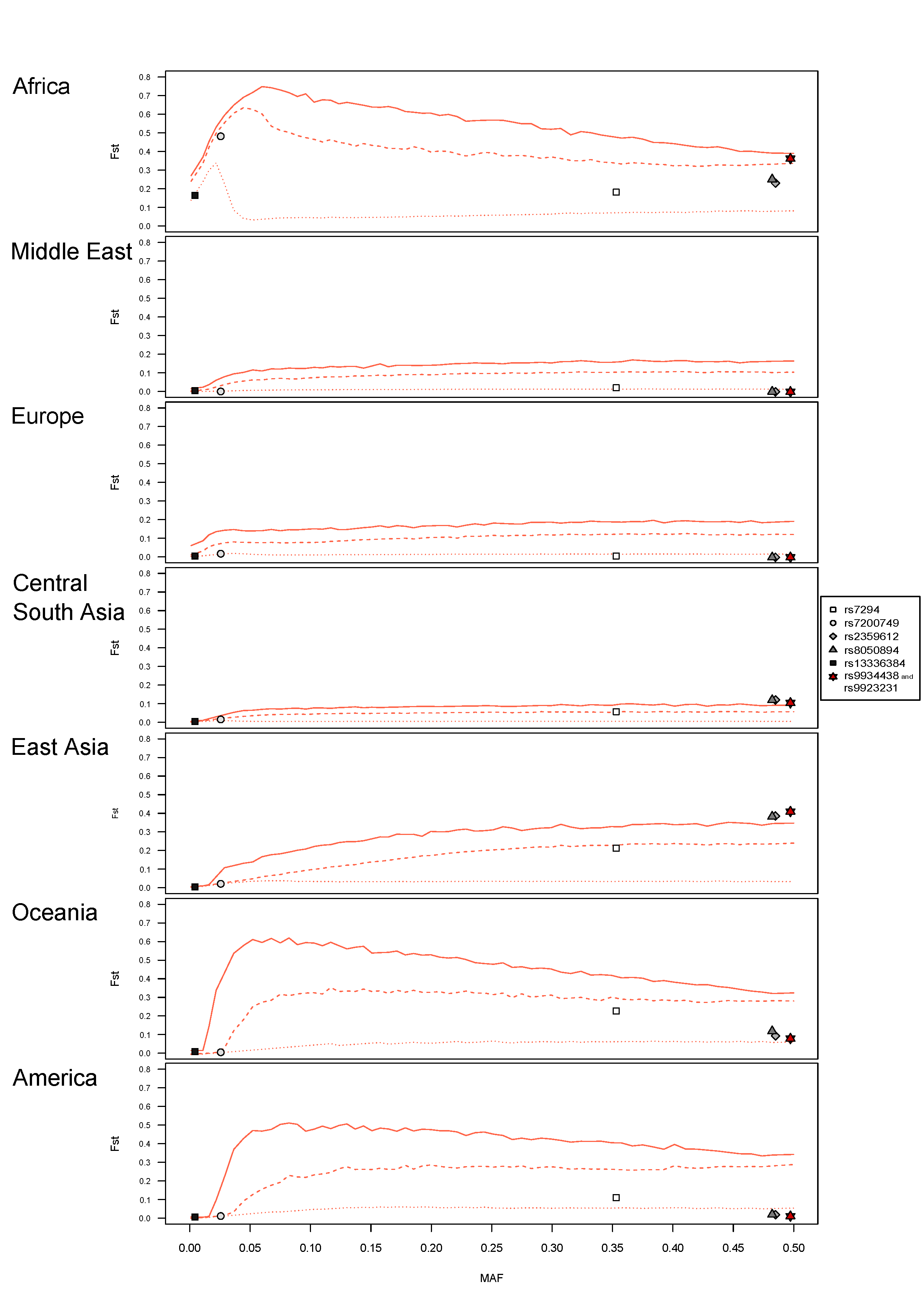

Supplement: Figure S3 — Genome-wide empirical distributions of inter-regional FST values against MAF in the seven geographic regions. Empirical distributions of FST were constructed by calculating an FST value for 644,413 SNPs having a MAF ≥0.001 at the global level. Individual values of FST calculated for each of the seven VKORC1 SNPs are plotted against their global MAF. The functional rs9923231 SNP is shown in red. The 50th, 95th and 99th percentiles are indicated as dotted, dashed and full red lines, respectively. (TIFF) [file pone.0053049.s003.tif]

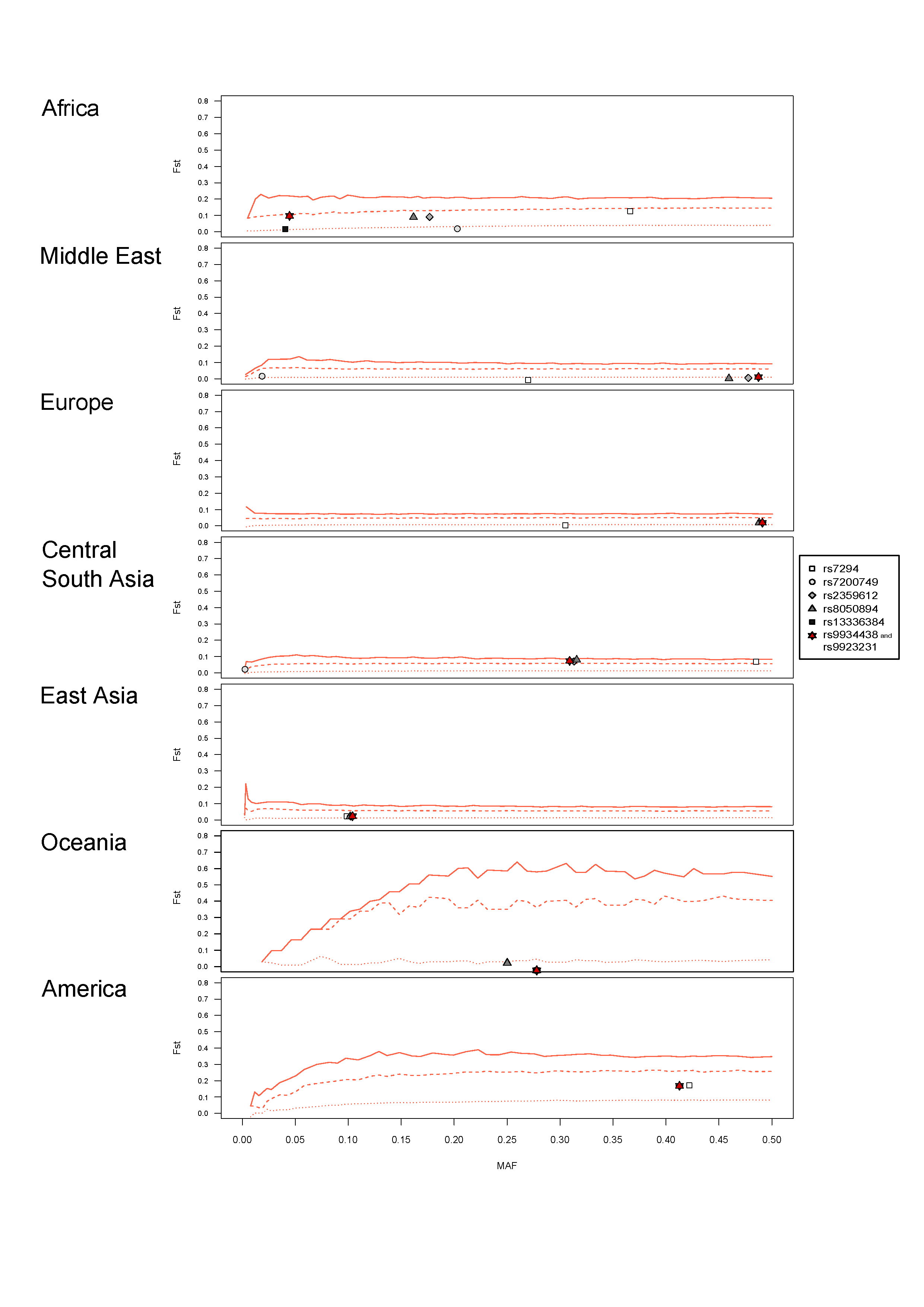

Supplement: Figure S4 — Genome-wide empirical distributions of intra-regional FST values against MAF in the seven geographic regions. Empirical distributions of FST were constructed by calculating an FST value for all SNPs having a MAF ≥0.001 at the intra-regional level. Individual values of FST calculated for each of the seven VKORC1 SNPs are plotted against the regional MAF. The functional rs9923231 SNP is shown in red. The 50th, 95th and 99th percentiles are indicated as dotted, dashed and full red lines, respectively. (TIFF) [file pone.0053049.s004.tif]

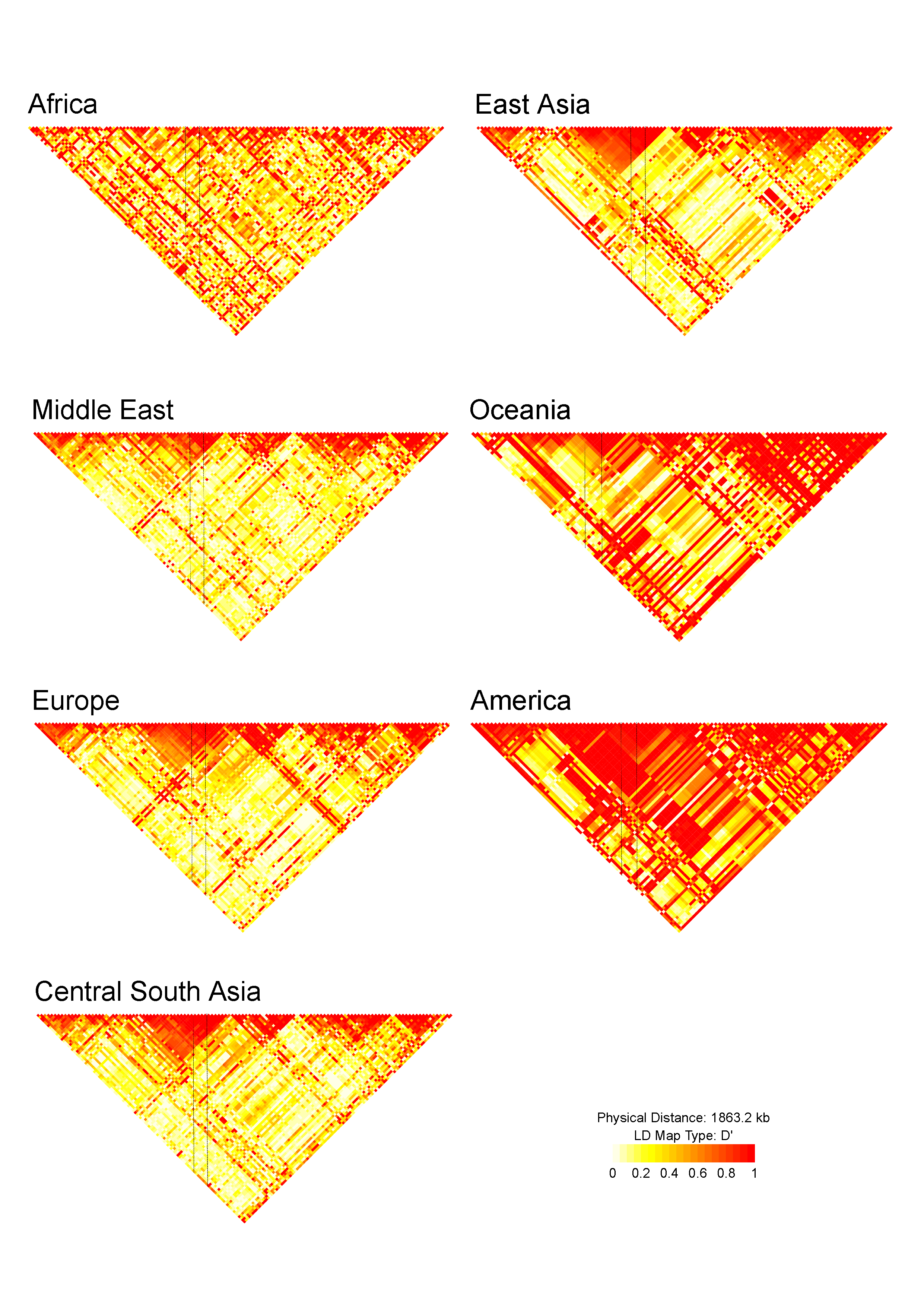

Supplement: Figure S5 — LD patterns over a 2 Mb region centered on VKORC1 in the seven geographic regions. Pairwise LD, depicted as D’, is shown for SNPs with a MAF ≥0.05 at the global level. D’ values are displayed in different colors from yellow to red for D’ = 0 to D’ = 1, respectively. The plot was produced using the snp.plotter R package [74]. The vertical dashed lines delineate VKORC1 gene position. (TIF) [file pone.0053049.s005.tif]

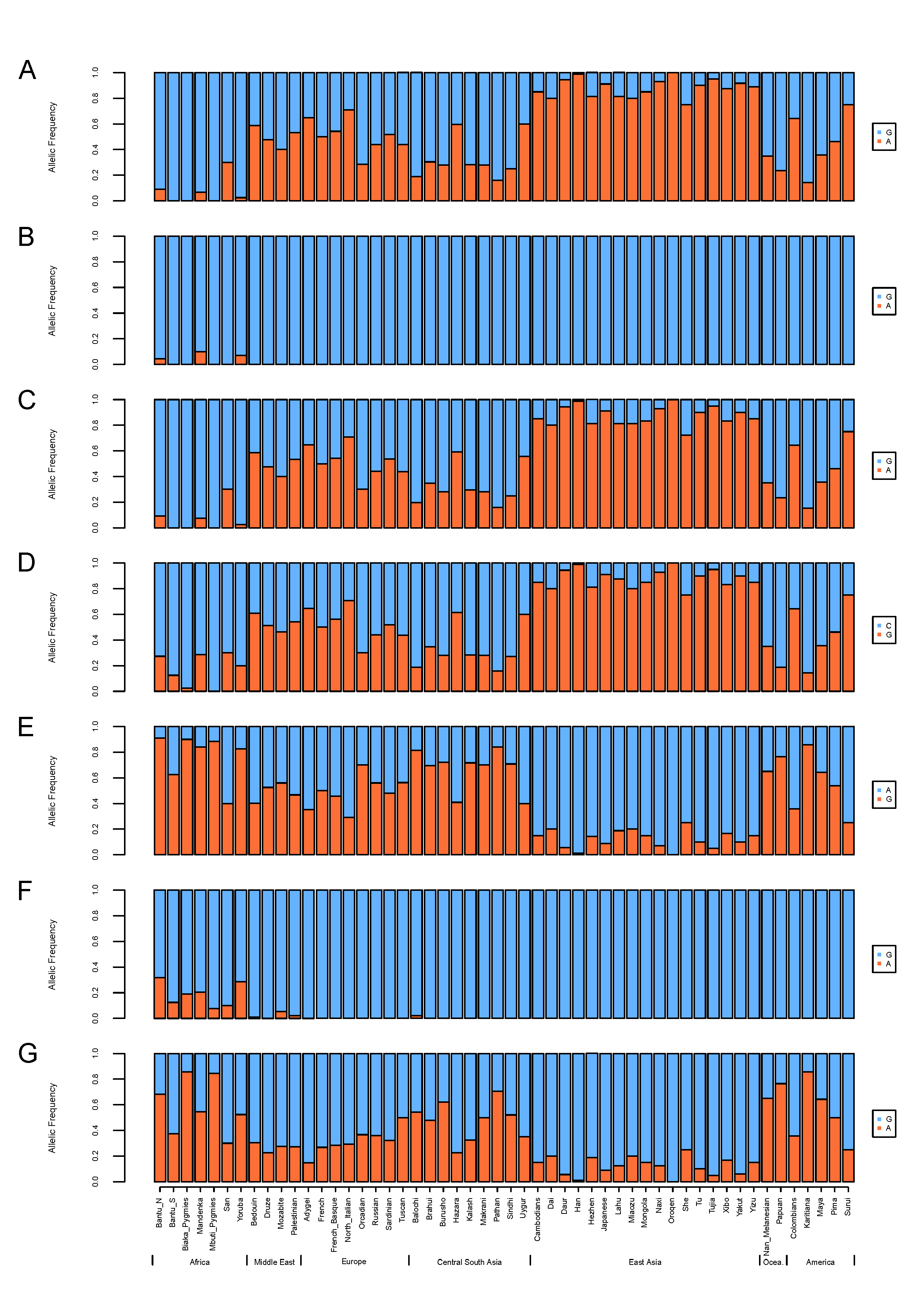

Supplement: Figure S6 — Allele frequency distribution of the seven VKORC1 SNPs in the 52 HGDP-CEPH samples: rs9923231 (A), rs13336384, (B) rs9934438 (C), rs8050894 (D), rs2359612 (E), rs7200749 (F) and rs7294 (G). The derived and ancestral alleles are represented in orange and blue, respectively. (TIF) [file pone.0053049.s006.tif]
